# A Rare Case of Bilateral Lateral Ventricular Subependymomas With Complete Resection of the Residual Tumor via Flexible Neuroendoscopy 20 Years After Initial Surgery

**DOI:** 10.7759/cureus.88975

**Published:** 2025-07-29

**Authors:** Yuta Nakagawa, Naoki Shinojima, Airi Miyazaki, Ken Uekawa, Hiroyuki Uetani, Toshinori Hirai, Yoshiki Mikami, Akitake Mukasa

**Affiliations:** 1 Department of Neurosurgery, Faculty of Life Sciences, Kumamoto University, Kumamoto, JPN; 2 Department of Diagnostic Radiology, Faculty of Life Sciences, Kumamoto University, Kumamoto, JPN; 3 Department of Diagnostic Pathology, Faculty of Life Sciences, Kumamoto University, Kumamoto, JPN

**Keywords:** flexible neuroendoscopy, intraventricular tumor, lateral ventricles, minimally invasive neurosurgery, subependymoma

## Abstract

Subependymoma is a benign, slow-growing tumor that arises from the ventricular wall. Although often asymptomatic, it can obstruct cerebrospinal fluid flow, leading to hydrocephalus. Most subependymomas are unilateral, typically located in the fourth ventricle, followed by the lateral ventricles. Bilateral involvement of the lateral ventricles is extremely rare.

We report the case of a 62-year-old man with bilateral subependymomas located in the anterior horns of both lateral ventricles, who presented with impaired consciousness due to epilepsy. Nineteen years earlier, the tumor in the anterior horn of the left lateral ventricle had been resected via craniotomy with frontal lobe uncapping. The lesion in the right lateral ventricle, initially small, gradually enlarged over two decades and was subsequently resected using flexible neuroendoscopy.

This approach allowed safe and effective tumor removal within a spacious intraventricular working environment while maintaining minimal invasiveness. In cases involving relatively small and hypovascular intraventricular tumors, flexible neuroendoscopy represents a viable minimally invasive surgical option. Continued technological advancements are anticipated to further enhance the safety and applicability of neuroendoscopic tumor resections.

## Introduction

Subependymoma is a slow-growing, benign tumor arising from the ventricular wall, classified as a World Health Organization (WHO) grade 1 central nervous system (CNS) neoplasm [1]. It most commonly occurs in middle-aged and elderly individuals [2,3]. Most subependymomas are asymptomatic and incidentally discovered on neuroimaging. When symptomatic, they typically present with signs of increased intracranial pressure due to hydrocephalus, such as headache, vomiting, and impaired consciousness, as well as seizures and motor deficits. The fourth ventricle is the most frequent site of origin, followed by the lateral ventricles. Subependymomas are typically solitary and unilateral; bilateral involvement of the lateral ventricles is exceedingly rare, with only a handful of cases reported in the literature to date [4-8].

According to the Brain Tumor Registry of Japan (2005-2008), subependymomas account for only 0.1% of all primary brain tumors [9]. The Central Brain Tumor Registry of the United States (CBTRUS) Statistical Report does not list subependymomas separately but includes them under non-malignant ependymal tumors, which constitute 0.7% of all CNS tumors, with an age-adjusted incidence rate of approximately 0.18 per 100,000 population per year [10].

Here, we report a rare case of bilateral subependymomas located in the anterior horns of both lateral ventricles. Of particular interest, the residual lesion was successfully resected using a flexible neuroendoscope two decades after the initial craniotomy. This case highlights not only the unusual bilateral presentation but also the long-term course and the potential of flexible neuroendoscopy as a minimally invasive approach for tumor removal within the ventricular system.

## Case presentation

A 62-year-old male patient with a history of poliomyelitis and a modified Rankin Scale (mRS) score of 1 at baseline was found unconscious at home 19 years ago and transported to a local hospital. At presentation, the Glasgow Coma Scale (GCS) score was 3 (E1V1M1), and the patient underwent endotracheal intubation and ventilator management. The level of consciousness gradually improved. Electroencephalographic data were not available, but the clinical course was consistent with non-convulsive status epilepticus, and anticonvulsant therapy was initiated, ultimately leading to surgical intervention.

**Figure 1:**
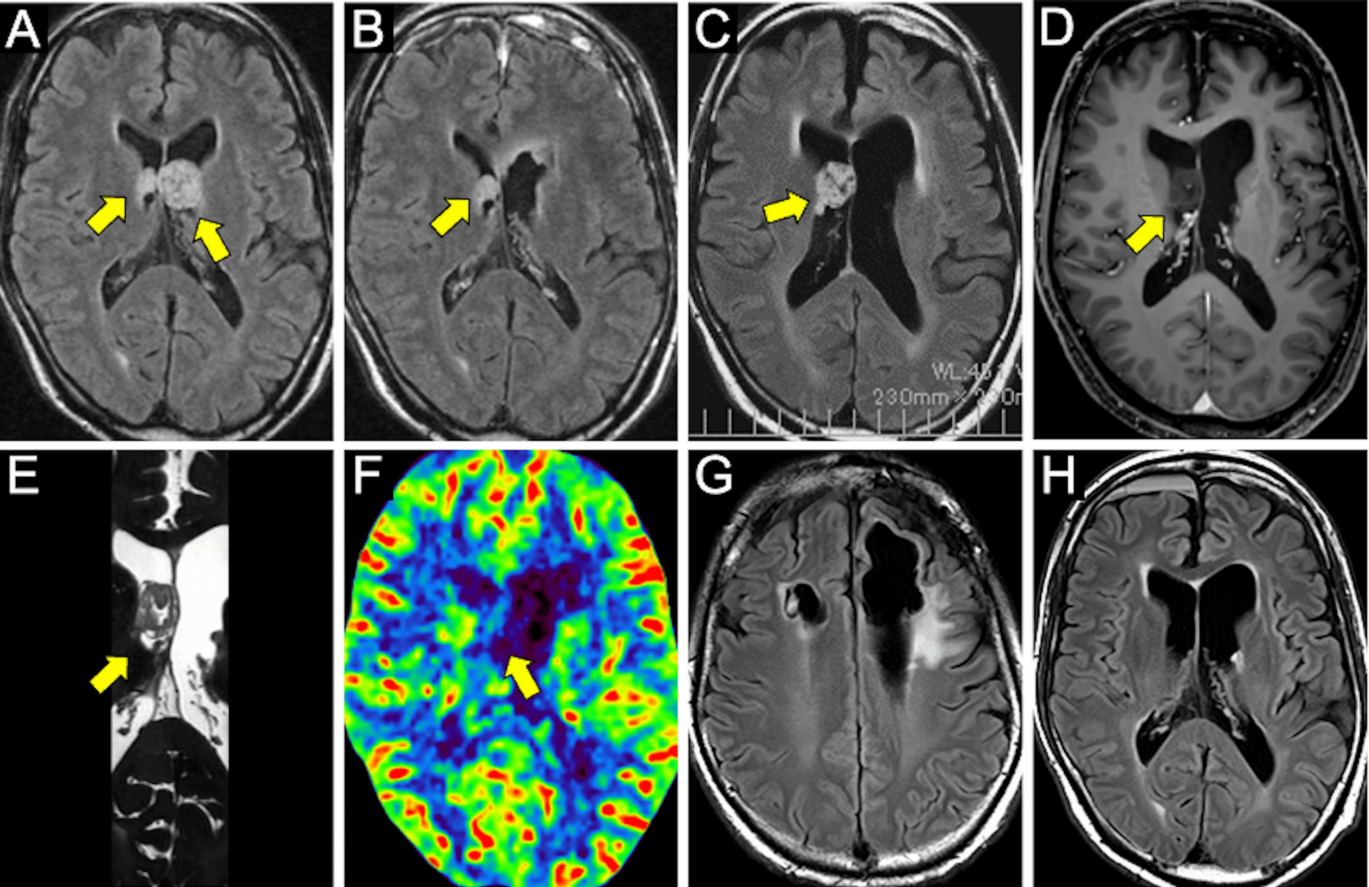
Images during the course of the disease A: FLAIR before initial surgery showing the tumor in the anterior horns of both lateral ventricles (arrow). B: FLAIR after the initial surgery showing the removal of the tumor on the left side and the persistence of the lesion on the right side (arrow). The images before the second surgery, FLAIR (C), Gadolinium contrast T1 (D), Heavy T2 (E), Perfusion CT (F) indicating that the tumor has grown compared to the previous examination (arrow); FLAIR 3 months after the second surgery showing uncapped left frontal lobe at initial surgery and narrow surgical corridor of right frontal lobe at the second surgery (G), no recurrence after gross total resection (H). FLAIR: fluid-attenuated inversion recovery

Magnetic resonance imaging (MRI) revealed neoplastic lesions in the anterior horns of both lateral ventricles. The lesion on the left measured 19 mm and obstructed the foramen of Monro, resulting in left ventricular enlargement. The lesion on the right measured 7 mm and was non-obstructive at the time (Figure 1A).

First surgery

The left intraventricular tumor was resected under the microscope via a conventional craniotomy using a left frontal transcortical approach with frontal lobe uncapping (Figure 1B). Histopathological analysis confirmed the diagnosis of subependymoma. The patient recovered without new neurological deficits.

Follow-up

Serial neuroimaging was performed over a 20-year period. No recurrence of the left-sided tumor was observed. However, the right-sided lesion gradually increased in size.

Second surgery

The patient was referred to our institution for the management of the enlarging right-sided tumor, which measured 23 mm on MRI (Figure 1C). The lesion exhibited no contrast enhancement (Figure 1D) and appeared to originate from the lateral aspect of the caudate nucleus, near the foramen of Monro (Figure 1E). CT perfusion showed no signs of hypervascularity (Figure 1F). Based on previous histopathology, subependymoma was the primary diagnosis; however, differential diagnoses included ependymoma and central neurocytoma.

**Figure 2:**
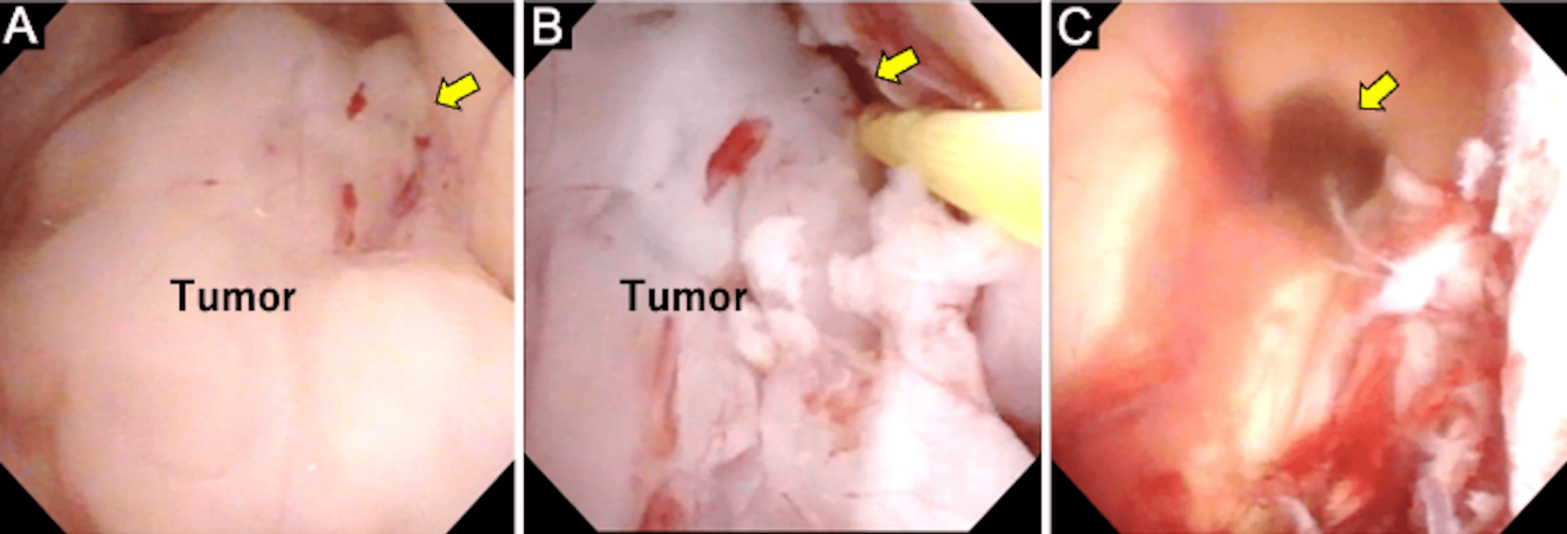
Intraoperative images A: Tumor located in the right lateral ventricle, attached to the caudate nucleus at the tumor attachment site (arrow). B: Approaching the tumor’s attachment site at the caudate nucleus and performing blunt dissection using biopsy forceps and a coagulation probe (arrow). C: After complete resection of the tumor, the foramen of Monro is visualized (arrow). The endoscopic viewpoint and viewing direction in this image are the same as those in panel A.

A small right frontal craniotomy was performed to allow for the possible use of a rigid endoscope; however, the procedure was ultimately completed using only a flexible neuroendoscope (Videoscope VEF TYPE V, Olympus, Tokyo, Japan) via a right frontal transcortical approach through a 17.5 Fr sheath (Medikit Co. Ltd., Miyazaki, Japan). The tumor was bluntly dissected from its attachment to the caudate nucleus using biopsy forceps and a coagulation probe in a fluid-filled environment with ventricles expanded by artificial cerebrospinal fluid (CSF) (Figures 2A, 2B). The feeding artery traversing the tumor was coagulated and transected using a RAF fiber probe (Muranaka Medical Instruments Co., Ltd., Bunkyō, Japan) with the Surgi-Max Plus system (Elliquence International, Baldwin, NY, USA). The tumor was completely resected (Figure 2C). The procedure lasted 7 hours with minimal blood loss (15 mL). The postoperative course was uneventful, with no neurological deterioration, hemorrhage, or hydrocephalus. The patient was discharged two weeks postoperatively with preserved preoperative function (mRS 1). Compared to the first surgery, the surgical corridor on the right side was much narrower (Figure 1G). The entire procedure was performed in a minimally invasive manner using only a flexible neuroendoscope. He remains recurrence-free more than two years after the second surgery (Figure 1H).

Histopathological findings

**Figure 3:**
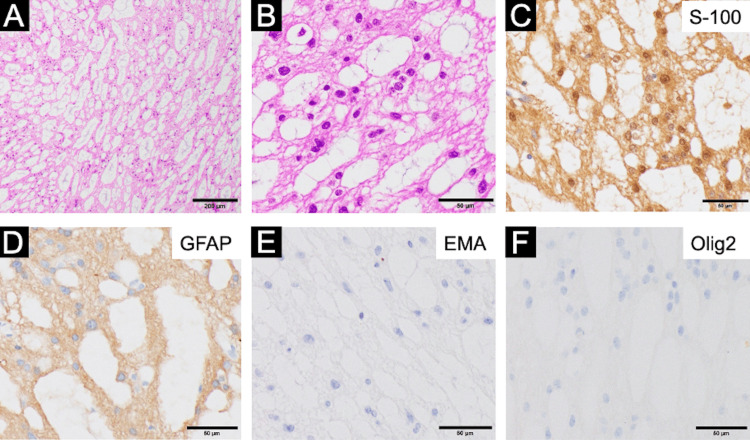
Histopathology Hematoxylin and eosin (HE) staining at (A) ×100 and (B) ×200; immunohistochemical staining for S-100 protein (C), glial fibrillary acidic protein (GFAP) (D), epithelial membrane antigen (EMA) (E), and oligodendrocyte transcription factor 2 (Olig2) (F) (all ×200)

Histological examination of the tumor removed during the second surgery revealed small tumor cells arranged in clusters within an abundant fine fibrillary matrix, along with microcystic structures. The tumor cells had sparse cytoplasm and showed minimal nuclear atypia (Figures 3A, 3B). Immunohistochemically, the tumor cells were positive for S-100 protein (Figure 3C), glial fibrillary acidic protein (GFAP; Figure 3D), and epithelial membrane antigen (EMA) with dot-like positivity (Figure 3E), and negative for oligodendrocyte transcription factor 2 (Olig2; Figure 3F). These findings were consistent with subependymoma, in line with the histopathological features of the left-sided tumor previously resected. The diagnosis was made according to the 2021 WHO Classification of Tumors of the Central Nervous System [1].

## Discussion

The etiology of bilateral subependymomas remains unclear, as most cases are solitary and unilateral. Bilateral involvement is exceedingly rare, with only a few cases documented in the literature to date, as summarized in Table 1 [4-8]. According to the Brain Tumor Registry of Japan (2005-2008), subependymomas account for only 0.1% of all primary brain tumors [9]. The CBTRUS registry classifies them under non-malignant ependymal tumors, comprising just 0.7% of all CNS tumors, with an age-adjusted incidence of 0.18 per 100,000 person-years [10].

**Table 1:** Summary of reported cases of bilateral lateral ventricular subependymomas, including the present case Clinical characteristics, tumor location, surgical approach, complications, and outcomes of previously published cases, along with the present case of bilateral lateral ventricular subependymomas. Abbreviations: Lt, left; Rt, right; yrs, years; GTR, gross total resection; NA, not applicable

	Age (yrs)	Sex	Presenting symptoms	Tumor location and size	Presence of hydrocephalus (unilateral/bilateral)	Surgical approach	Laterality of resection (simultaneous or staged)	Interval between resections (if not simultaneous)	Surgical complications (if any)	Outcome (survived or deceased)	Recurrence (yes/no)	Overall survival (months/ years)
Case 1 (Miguel Y et al. [4])	69	Female	Headache, ataxia, apraxia, right-sided weakness, and neglect	In the posterior body, the occipital horn and the trigone of both right and left ventricles, with extension to the left corona radiata. Lt: 25x27x37 mm; Rt: unknown.	No	GTR via left parietal occipital craniotomy	The resection of the left-sided lesion.	no	no	survived	no	unknown
Case 2 (Rath TJ et al. [5])	20	Male	Severe headache and altered mental status	In the right and left lateral ventricles. The size is unknown.	Bilateral	Right frontal craniotomy with an interhemispheric transcallosal approach	simultaneous	no	no	survived	no	unknown
Case 3 (Kumar R et al. [6])	25	Male	Symptoms of raised intracranial pressure	In the bilateral occipital region. The size is unknown.	No	unknown	staged	6 weeks	no	survived	no	3 years
Case 4 (Moinuddin FM et al. [7])	48	Male	Unknown	In both anterior horns of the lateral ventricles. The size is unknown.	No	Endoscopic biopsy was performed via a left frontal burr hole.	simultaneous	no	no	survived	no	8 months
Case 5 (Minh ND et al. [8])	40	Male	Bilateral parietal-occipital headache and dizziness	At the trigone and the occipital horn of the bilateral lateral ventricles. Lt: 43x21x25 mm; Rt: 42x18x19 mm	No	Partial resection surgery for the left ventricular tumor.	simultaneous	no	postoperative paraventricular edema	survived	no	unknown
Present case	62	Male	Impaired consciousness due to epilepsy	Lt: 19 mm → no recurrence; Rt: 7→23 mm over 20 yrs	First: Lt. hydrocephalus; Second: no hydrocephalus	First: Microscopic GTR via left frontal transcortical approach; Second: Flexible neuroendoscopic GTR via right frontal narrow corridor	staged	19 yrs	no	survived	no	21 years

Asymptomatic subependymomas are generally managed conservatively with regular imaging follow-up [2,3]. However, when progressive growth occurs, as in this case, especially with the risk of hydrocephalus, surgical resection should be considered. Given the typically indolent nature of subependymoma, a minimally invasive strategy is preferable to avoid iatrogenic complications. In this case, the left-sided tumor was resected via conventional craniotomy 19 years earlier. In contrast, the right-sided tumor was removed through a small craniotomy using a flexible neuroendoscope alone, within a narrow transcortical corridor. This approach enabled safe resection without rigid instruments.

Rigid endoscopic and microscopic surgeries are performed in a dry field, where the ventricles collapse due to CSF drainage, resulting in a deeper and narrower working space. This often necessitates a wider and more invasive surgical corridor. Conversely, flexible neuroendoscopy is conducted in a fluid-filled ventricular system expanded with artificial CSF. This provides a spacious working environment and improved anatomical orientation. The flexible tip allows multi-angled manipulation, enhancing visualization and reducing the risk of disorientation.

However, this technique is not without limitations. Flexible neuroendoscopes typically contain a single working channel, precluding the simultaneous use of multiple instruments. This restricts the surgeon’s ability to perform traction and coagulation simultaneously, potentially prolonging operative time. Additionally, bleeding control is more challenging compared to rigid approaches.

Despite these limitations, flexible neuroendoscopy remains a valuable tool in the resection of small, hypovascular intraventricular tumors. Several reports, including the present case, demonstrate its feasibility and safety [11]. Future advancements may include the development of multi-channel neuroendoscopes and integration with robotic systems that offer improved maneuverability and simultaneous instrument deployment.

This case not only underscores the rarity of bilateral subependymomas but also illustrates the long-term evolution of such tumors and the utility of flexible neuroendoscopy for safe and minimally invasive resection.

## Conclusions

Although subependymomas occasionally occur at multiple sites within the ventricular system, bilateral involvement of the lateral ventricles remains exceedingly rare. Cases successfully resected using a flexible neuroendoscope alone are even more uncommon. This case illustrates that, in appropriately selected patients with small, hypovascular intraventricular tumors, flexible neuroendoscopy can offer a safe and effective minimally invasive surgical option. Further innovation in neuroendoscopic technology is anticipated to broaden its application and safety in neurosurgical practice.
